# EDA-Containing Fibronectin Increases Proliferation of Embryonic Stem Cells

**DOI:** 10.1371/journal.pone.0080681

**Published:** 2013-11-14

**Authors:** Noelia Losino, Ariel Waisman, Claudia Solari, Carlos Luzzani, Darío Fernández Espinosa, Alina Sassone, Andrés F. Muro, Santiago Miriuka, Gustavo Sevlever, Lino Barañao, Alejandra Guberman

**Affiliations:** 1 Laboratorio de Regulación Génica en Células Madre, Departamento de Química Biológica, Facultad de Ciencias Exactas y Naturales, Universidad de Buenos Aires (UBA), Ciudad de Buenos Aires, Argentina; 2 Instituto de Química Biológica - Ciencias Exactas y Naturales (IQUIBICEN), UBA/Consejo Nacional de Investigaciones Científicas y Técnicas (CONICET), Ciudad de Buenos Aires, Argentina; 3 Laboratorio de Biología del Desarrollo Celular, Fundación para la Lucha contra las Enfermedades Neurológicas de la Infancia (FLENI), Buenos Aires, Argentina; 4 International Centre for Genetic Engineering and Biotechnology, Trieste, Italy; 5 Departamento de Fisiología y Biología Molecular y Celular, Facultad de Ciencias Exactas y Naturales, Universidad de Buenos Aires, Ciudad de Buenos Aires, Argentina; Indian Institute of Toxicology Reserach, India

## Abstract

Embryonic stem cells (ESC) need a set of specific factors to be propagated. They can also grow in conditioned medium (CM) derived from a bovine granulosa cell line BGC (BGC-CM), a medium that not only preserves their main features but also increases ESC´s proliferation rate. The mitogenic properties of this medium were previously reported, ascribing this effect to an alternative spliced generated fibronectin isoform that contains the extra domain A (FN EDA^+^). Here, we investigated if the FN EDA^+^ isoform increased proliferation of mouse and human ES cells. We analyzed cell proliferation using conditioned media produced by different mouse embryonic fibroblast (MEF) lines genetically engineered to express FN constitutively including or excluding the EDA domain (FN EDA^-^), and in media supplemented with recombinant peptides containing or not the EDA. We found that the presence of EDA in the medium increased mouse and human ESC’s proliferation rate. Here we showed for the first time that this FN isoform enhances ESC’s proliferation. These findings suggest a possible conserved behavior for regulation of ES cells proliferation by this FN isoform and could contribute to improve their culturing conditions both for research and cell therapy.

## Introduction

Embryonic stem cells (ESC) are derived from the inner cell mass of blastocysts and have the potential to give rise to all cell types of the body. This property, named pluripotency, is restricted only to a few types of stem cells. Pluripotent cells provide a powerful model to investigate molecular and cellular processes involved in lineage-specification and embryogenesis, to perform drug screening, and to assess potential applications in the field of tissue engineering and cell therapy.

Many factors and signaling pathways affect pluripotent stem cells’ proliferation, like MAPK, phosphoinositide 3-kinase [1,2] and glycogen synthase kinase 3 pathways [3], mTor [4], BMP-4 and Wnt1 [5], brain natriuretic peptide signaling [6], among others. Culture conditions like seeding density [7], oxidative stress [8] and nutrient availability, also influence stem cell propagation. It has been reported that high glucose concentrations present in the culture medium induce fibronectin (FN) expression in mESC, and that this molecule could be responsible for the augmented proliferation in response to the high glucose concentrations [9,10]. 

We have recently shown that conditioned medium (CM) from a bovine granulosa cell line (BGC-CM) is able to maintain mouse pluripotent stem cells’ self-renewal, including ESC and induced pluripotent stem cells (iPSC), while preserving their unique properties, in culture without Leukemia Inhibitor Factor (LIF) addition [11,12]. Pluripotent stem cells growing on BGC-CM expressed stem cell markers and remained pluripotent. Moreover, we also found that mES cells cultured in these conditions have an increased proliferation rate compared with cells cultured in ESC standard proliferation medium (PM) [12]. The conditioner cell line was previously established [13] and formerly selected by its mitogenic properties on the same granulosa cell line and on primary cultures [14]. It was reported that a form of FN that alternatively includes spliced domain A (EDA) (FN EDA^+^) present in the above mentioned CM, could be responsible for the mitogenic effect. The authors showed that FN-depleted conditioned medium did not exhibit proliferation stimulatory effect on granulosa cells, and that supplementation of this CM with plasma FN, which lacks exon EDA, had also no effect on cells mitogenic properties [15]. It is worth mentioning that FN EDA^+^ is usually expressed in proliferating tissues, suggesting that this isoform may play a role in cell proliferation [15–18]. Moreover, it was shown that EDA inclusion potentiated the ability of FN to promote cell cycle progression [19].

Considering all these evidences, in this work we studied the effect of FN EDA^+^ on ESC proliferation. We found that this specific isoform is capable of augmenting the mitogenic capabilities of both mouse and human ES cells. These findings suggest a possible conserved mechanism for regulation of ES cells proliferation by this FN isoform. 

## Materials and Methods

### Cell culture

The E14-derived Ainv15 and R1 mESC lines were obtained from ATCC and cultured as previously described [11,12,20]. 

The human embryonic stem cell (hESC) line WAO9 was purchased from WiCell Research Institute, and the hESC line HUES-5 was acquired from Harvard University and the Howard Hughes Medical Institute at low passages (p15 to p20) [21]. The hESC lines were maintained on a mitotically inactivated MEF feeder layer in medium comprised of Dulbecco's Modified Eagle's Medium/Ham's F12 supplemented with KSR 20% 2 mM nonessential amino acids, 2 mM L-glutamine, 100 U/ml penicillin, 50 μg/ml streptomycin, 0.1 mM β-mercaptoethanol and 4 ng/ ml of bFGF on diluted (1/40) Matrigel^TM^ coated dishes in MEF conditioned medium. For the conditioning medium, 3×10^6^ inactivated MEF cells were incubated for 24 h with 25 ml of DMEM/F12 medium supplemented with 5% KSR and 2 ng/ml of bFGF (in addition to the other aforementioned supplements) and stored at -20 °C. After thawing, fresh aliquots of KSR and bFGF were added to the medium to render a final concentration of 20% and 4 ng/ml, respectively. *In vitro* differentiation protocol was performed as previously described [22] from ES cells cultured on Matrigel for three days in the presence or absence of exogenous EDA.

### Conditioned Media obtention

MEF were prepared from EDA^wt/wt^, EDA^+/+^, and EDA^-/-^ animals, described previously [23]. Briefly, MEF were obtained from 13.5 days embryos, and propagated at high density in DMEM high glucose supplemented with 10% FBS (GIBCO), glutamine, and antibiotics for successive passages until MEF cell lines were established. Then, MEF cell lines were cultured in the same culture medium to 80% confluence. Culture medium was replaced by fresh medium, and incubated for 24 hours. After incubation, conditioned medium was removed, centrifuged at 2,000 x g during 10 minutes and supplemented with LIF, 5% FBS, nonessential amino acids and 0.5 mM beta-mercaptoethanol before use. 

### Cell proliferation assay

In proliferation assays, ES cells were plated on feeder free conditions. Human ES cells were plated on Matrigel coated plates with MEF conditioned medium and mouse ES cells on gelatin coated plates with standard proliferation medium with LIF. Twenty four hours later, the media were replaced by the corresponding medium for each experimental condition.

For crystal violet or MTT assays, mouse ES cells were seeded in a flat bottom 96-well plate. For each experiment, 3,000 cells per well were seeded on gelatin coated plates in standard proliferation medium. 24 hours later, the medium was removed and replaced by the corresponding medium. Cell proliferation was quantified by the MTT method (SIGMA) or by crystal violet staining (Anedra), according to manufacturer’s instructions. Briefly, for MTT method, culture media were prepared without phenol red. A volume of MTT solution equal to 20% of culture medium was added 3 hours prior to measurement. Formation of colored product, the orange formazan derivative, was quantified collecting the culture medium, reading its absorbance at 450 nm and subtracting background contribution from the reactive sodium salt of MTT, at 690 nm in a multiwell plate reader (Microplate Reader, BioRad model 680). An initial measurement was taken 3 hours after seeding cells in the plate. Afterwards, absorbance at 450 and 690 nm was measured once a day during at least 5 days. In order to assess cell proliferation rate, corrected absorbance vs. time were graphed and slopes for each culture medium were then compared. For crystal violet staining, cells were rinsed twice with ice-cold PBS and then fixed with methanol for 10 minutes at -20 °C. Staining was carried out with 0.5 % crystal violet in methanol for 10 minutes and then washed with water until the dye was completely removed. Plates were let dry at room temperature and crystal violet was solubilized with 10 % acetic acid. Absorbance was measured at 590-595 nm. 

For the wound healing assay, hES cells were plated on Matrigel coated plates with MEF conditioned medium. After 24-48 hours, a “wound gap” was created in the center of the hESC’s colonies by scratch or aspiration of the cells with a 200 μl pipet tip. The cells were rinsed with PBS and the media was replaced by fresh MEF conditioned medium containing or not the indicated peptides. The healing was monitored by microphotograph every day. The images were analyzed with the ImageJ software (ImageJ 1.44p, Wayne Rasband, National Institutes of Health, USA. http://imagej.nih.gov/ij, Java 1.6.0_20 (32-bit).

To study Bromodeoxy Uridine (BrdU) incorporation, BrdU immunostaining was used to identify proliferating cells. After 72 hours of treatment as indicated, the samples were pulsed with 100 μM BrdU in the corresponding medium for 30 minutes or 15 minutes for human or mouse ES cells, respectively; ﬁxed in 4% paraformaldehyde for 30 minutes, and permeabilized with 0.5% Triton X-100 for 10 min or 10 minutes in methanol -20 °C, respectively. Samples were then incubated in 2 M HCl for 30 minutes at 37°C, and then washed in PBS containing 0.2% Tween 20. The samples were incubated overnight at 4°C with primary antibody against BrdU (GE Healthcare, Cat. RPN202), washed with PBS and then were incubated during 1 hour with the secondary antibody (anti-mousse IgG FITC, Sigma, # F0257) and DAPI. Then, the samples were washed with PBS, mounted in Mowiol (Calbiochem, Cat# 475904) and take microscope images in an Olympus IX71 fluorescence microscope. For Ki67, the same immunostaining protocol was used without the HCl incubation. The primary antibody was Novocastra Leica: NCL-L-Ki67-MM1.

### Production of EDA-containing and EDA-lacking recombinant peptides

EDA-containing and EDA-lacking recombinant peptides were obtained as described [24]. Briefly, we used the previously BL21 *E. coli* competent cells transfected with 804 bp containing the EDA segment flanked by the 11th and 12th type III repeats, or 534 bp lacking the EDA domain cloned in the BamHI site of the pProEX Htb expression vector. Peptides expression was induced by addition of isopropyl-β-D-1-thiogalactopyranoside to a final concentration of 1 mM. Cells were then lysed and disrupted by freezing/thawing and sonicated in a BRANSON - Fisher Scientific Model 500 sonicator, 8 pulses of 10 seconds at 15% amplitude, with 10 seconds intervals, on ice. Protein extracts were loaded on a 12% SDS-PAGE gel. Bands corresponding to the EDA-containing and EDA-lacking peptides were detected by staining the gel with 1 M KCl, cut out from the gel and electroeluted in 50 mM carbonate-bicarbonate buffer, pH 9.6, during 3 hours at 150 mA in a benzoylated dialysis tubing with a 2000 MW pore size (Sigma, D-2272). Protein concentration was determined by Bradford Protein Assay. We performed a dose-response curve to determine the most effective dose (Figure S1). We observed a dose-dependent effect in the mES cells proliferation rate. Then, we selected the dose named “dilution 1”, corresponding to 10 µg of proteins per ml of medium, to perform the next series of experiments. All the experiments were performed with the same batch for each peptide.

### Statistical analysis

An unpaired t-test for unequal variances was applied to determine differences between proliferation levels of ES cells in the presence of EDA^+/+^ MEF-CM or EDA^-/-^ MEF-CM. Normality was assessed by Shapiro-Wilk test. A One way ANOVA was used to determine differences between proliferation levels of ES cells in the presence of medium supplemented with EDA-containing or EDA-lacking recombinant peptides for 2 and 3 days. Normality was assessed as mentioned before and variance homogeneity assumption was assessed by Levene test. Whenever those assumptions were not accomplished, data were transformed. In all cases, statistical significance was assumed when *P* ≤ 0.05. A Chi Square test was used to compare the number of positive Ki67 cells and positive BrdU cells between control and each treatment. Bonferroni´s method was used to correct p values for multiple comparisons. The data were analysed using SPSS version 17 (SPSS Inc, Chicago, IL). 

### Teratoma formation assay

Teratoma formation assay and analysis was performed as previously described [12] from Ainv15 mouse ES cells cultured for three days on gelatin coated plates in the presence or not of exogenous EDA.

### Reverse transcription-polymerase chain reaction

RT-PCR were performed and analyzed as previously described. Primer sequences were supplied in the same references 11,12,20, except for mouse and human FN EDA+ and human Cardiac Troponin, that are indicated in Table S1.

## Results

We previously reported that conditioned medium (CM) from a bovine granulosa cell line (BGC-CM) is able to maintain mES cells’ and iPSCs’ self-renewal and pluripotency. Moreover, BGC-CM increased their proliferation rate [12]. Based on evidences involving FN EDA^+^ as the factor present in BGC-CM responsible for the increased proliferation in granulosa cells [15], we hypothesized that this FN isoform might also be mitogenic for ES cells.

To test this possibility, we reasoned that addition of the FN EDA^+^ isoform to the culture medium should mimic the mitogenic effects of BGC-CM. To this end, we cultured the E14-derived ESC line, Ainv15, in different culture contexts providing the two different FN variants, FN EDA^+^ or FN EDA^-^, and then studied ESC’s proliferation by crystal violet staining or MTT assay. For this analysis we designed two different experimental approaches.

**Figure 1:**
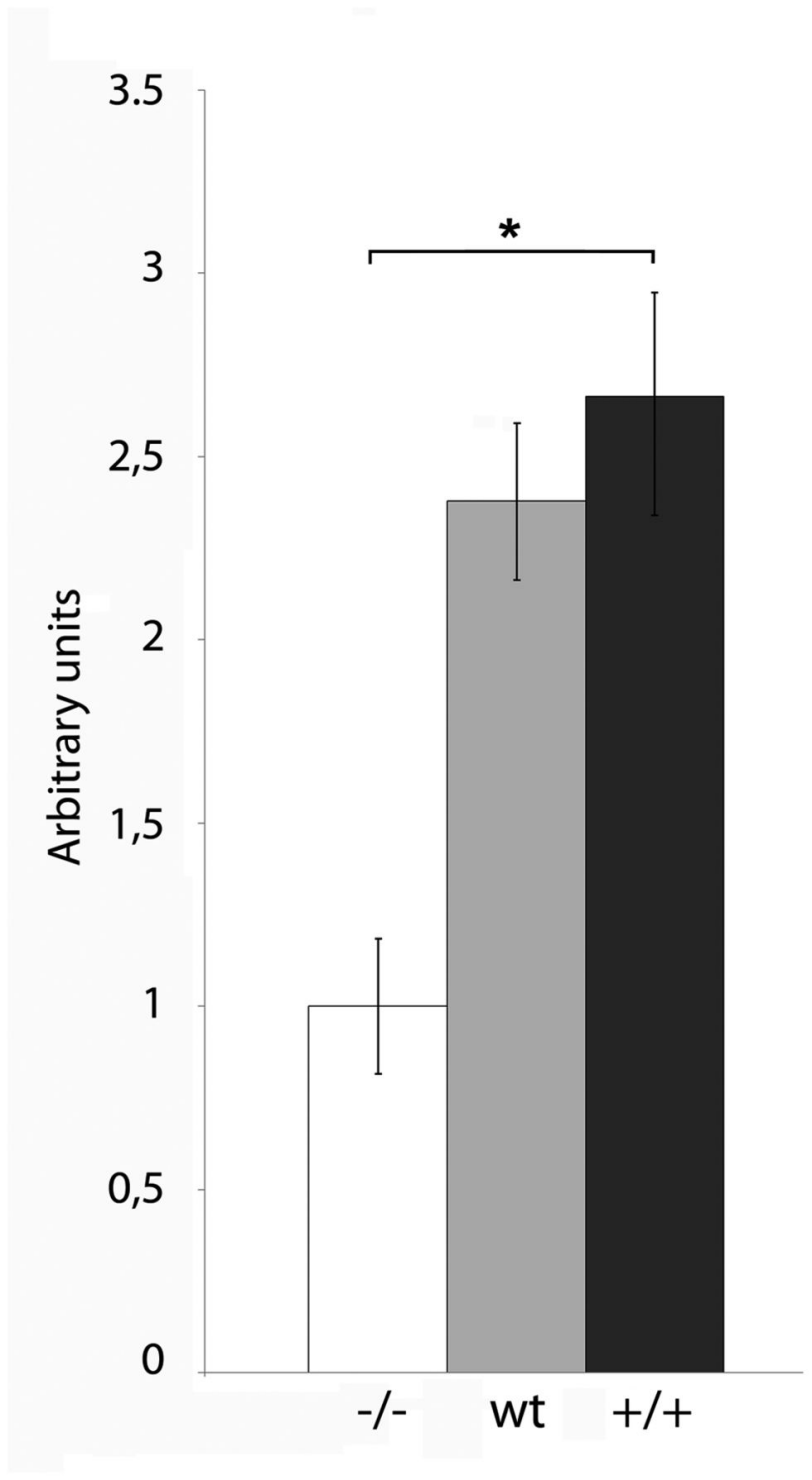
MEF conditioned medium that contains FN EDA^+^ increases the proliferation rate of mESC. Ainv15 mES cells were cultured in the media conditioned by wild type MEF (wt), EDA^+/+^ MEF (^+/+^) or EDA^-/-^ MEF (^-/-^) for 2 days. The proliferation was evaluated by MTT assay. A representative experiment of at least three replicates is shown. Bars represent mean ± SD. Statistical analysis was done by an unpaired t-test. * indicate significant differences between treatments (*P* < 0.01).

In the first approach, we cultured mES cells in the presence of media conditioned by three different MEF lines. Two of them were obtained from genetically modified mice, whose FN genes were engineered to express only one of the FN isoforms respect to EDA inclusion [23]. EDA^+/+^ mice have optimized 5’ and 3’ EDA splicing sites and, thus, generate FN mRNA with constitutively included EDA exon [25]. On the contrary, EDA^-/-^ mice have the EDA exon deleted and produce FN mRNA constitutively lacking it [23]. We also used MEF from wild type mice as a control. Therefore, the media conditioned by these different MEF lines (MEF-CM) are expected to contain different FN isoforms. MEF derived from EDA^-/-^ mice express solely the FN isoform that excludes EDA (FN EDA^-^), wild type MEF produces both forms, 60% EDA+, 40% EDA-, approximately [23], and the EDA^+/+^ MEF line expresses only the isoform that includes EDA [23,24]. The cells were plated on feeder free gelatin coated plates in the standard proliferation medium and 24 hours later, when the cells were attached, the medium was changed. Colony morphology and proliferation were monitored daily. As shown in Figure 1, when the mESC were cultured in EDA^-/-^ MEF-CM, their proliferation rate was considerably lower than when they were propagated in *wt* MEF-CM or in EDA^+/+^ MEF-CM. Although we didn´t find important differences in the proliferation rate of mES cells when cultured with *wt* MEF-CM and in EDA^+/+^MEF-CM, we noticed a trend showing a slight increase in the proliferation rate of the latter. When comparing proliferation rate between ES cells cultured in EDA^+/+^ MEF-CM or EDA^-/-^ MEF-CM we found significantly differences. These results suggest that the FN EDA^+^ present in the conditioned media is a mitogenic factor for ESC.

**Figure 2:**
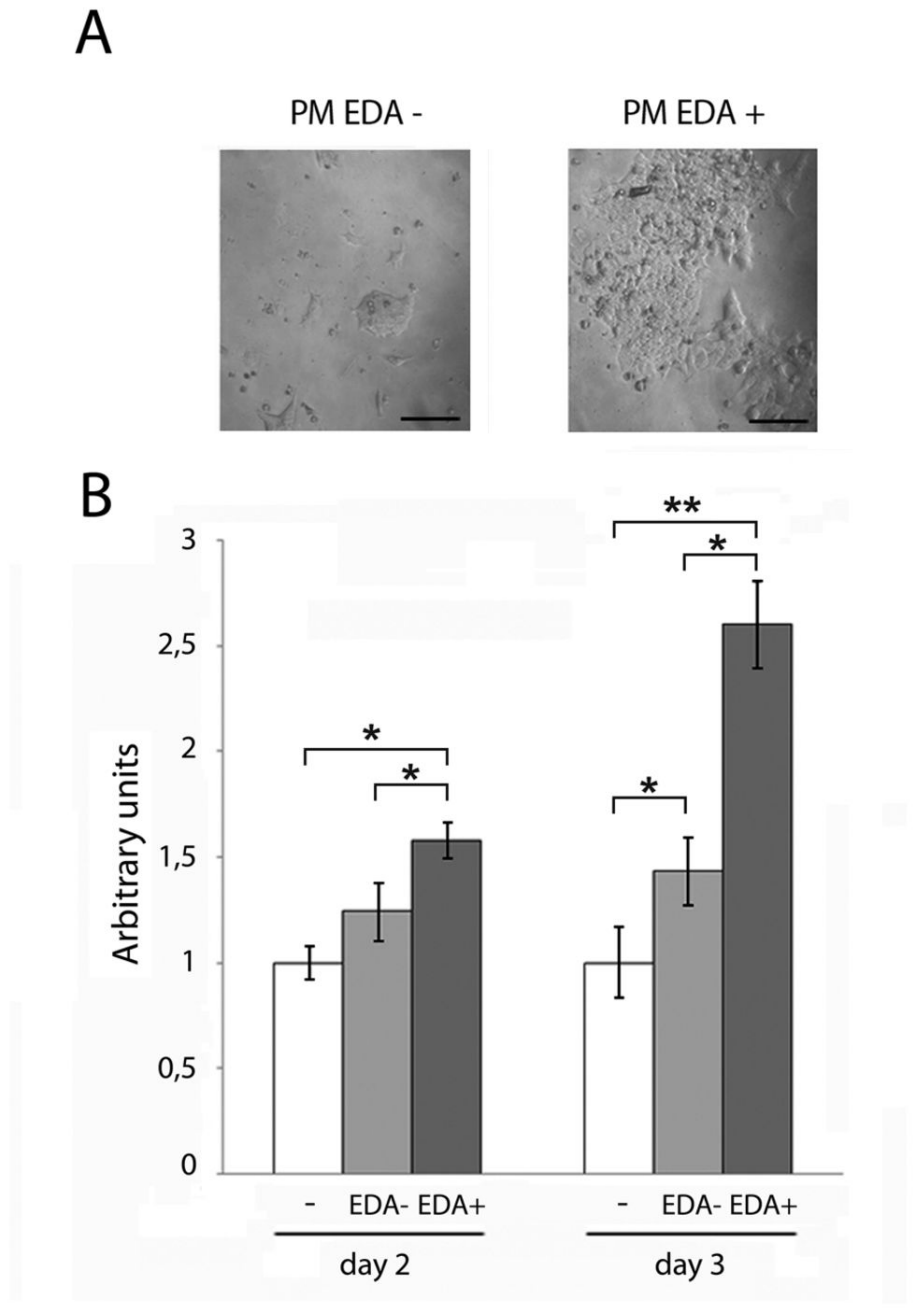
EDA^+^ but not EDA^-^ peptide increases the proliferation rate of mESC. (A) Ainv 15 mouse ES cells were cultured in the standard media supplemented by the corresponding peptide preparation to a final dose of 10 µg of protein per ml of medium for 72 hours. (EDA-containing recombinant peptide, EDA^+^; EDA-lacking recombinant peptide, EDA^-^). Representative brightfield pictures are shown. Bars correspond to 50 µm. (B) The proliferation of Ainv15 mES cells cultured in the media described in B for 48 or 72 hours, as indicated, was evaluated by crystal violet. A representative experiment of at least three replicates is shown. Bars represent mean ± SD. Statistical analysis was done by One way ANOVA with Bonferroni test for multiple comparisons. * indicate significant differences between treatments (*P* < 0.05); ** indicate significant differences between treatments (*P* < 0.005).

To confirm these results and further determine whether the EDA segment was capable of increasing ESC’s proliferation rate, we next assessed ESC’s proliferation in standard proliferation medium supplemented with recombinant peptides containing or not the EDA [24]. We first performed a pilot experiment to determine the most effective dose, as described in Materials and Methods section (Figure S1). mESC were cultured for 48 or 72 hours in proliferation medium supplemented with recombinant peptides containing or not the EDA. As shown in Figure 2, ES cells’ proliferation rate augmented significantly more in the presence of EDA^+^ peptide than in the presence of EDA^-^ peptide. No significant differences were detected between untreated cells and cells treated with EDA^-^ peptide, at 48 hours. The proliferation rate of cells supplemented with the EDA^+^ peptide was significantly higher than cells that were supplemented with EDA^-^ or no peptide. This difference was even higher at 72 hours.

To further confirm these results, we analyzed BrdU incorporation, a structural analogue of nucleotide Thymidine that is incorporated into DNA during its replication in actively proliferating cells. We compared the number of BrdU positive cells among untreated or EDA-treated mES cells. In this experiment, we cultured mES cells in the presence of EDA+ peptide or in EDA^+/+^ MEF-CM. As shown in Table 1, EDA treatment increased the number of BrdU positive cells.

**Table 1:** Analysis of BrdU incorporation in Ainv 15 mES cells in different media conditions.

	**Positive BrdU cells (%)**	N^(1)^	**p value** ^(2)^
**Control**	58,70	477	
**EDA+/+ MEF-CM**	68,84	337	0.01
**FN EDA+ Peptide**	87,69	463	<0.001

(1) Number of total cells positive for DAPI staining (2) p values resulted from comparison of each treatment with the control. A Chi Square test was used to compare the number of positive BrdU cells between control and each treatment. Bonferroni´s method was used to correct p values for multiple comparisons. Control: untreated cells. Values correspond to a representative experiment with three biological replicates.

 As a whole, these results demonstrate that FN EDA^+^, both secreted by MEF lines or added to the medium as recombinant peptide, increases the proliferation rate of mES cells.

**Figure 3:**
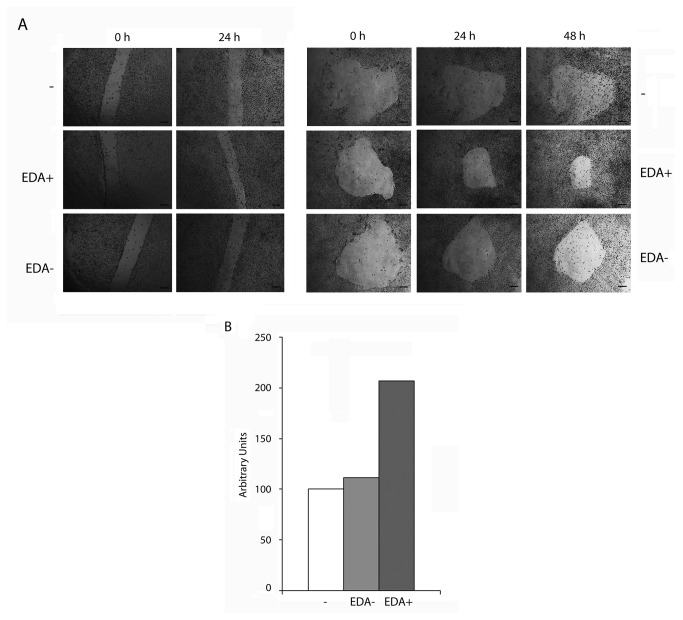
FN EDA^+^ but not FN EDA^-^ increases the proliferation of human ESC. WAO9 human ES cells were plated in standard proliferation medium. 24 hours later, when the cells were attached, medium was replaced by fresh medium containing the corresponding peptide preparation to a final dose of 10 µg of protein per ml of medium. (A) Representative brightfield pictures of the wound-healing assay. The scratch was made the same day that the peptide was added to the medium. Left panel, linear scratch; right panel, circular scratch. The references are the same than in Figure 2. Bars correspond to 200 µm. (B) Quantification of the filled areas of a representative experiment of three replicates. Proliferation was calculated from the decrement in damaged area. The areas were quantified with the ImageJ software. The filled area in each condition was referred as the amount of filled area in the control, considered as 100%. The graph is representative of a scratch wound healing assay of three replicates.

To extend our analyses, we finally studied the effect of EDA^+^ and EDA^-^ peptides on WAO9 human ES cells. We first analyzed cell proliferation by two different modalities of the wound-healing assay: by performing a linear or a circular wound to the monolayer [26,27] and supplementing the culture medium with the recombinant peptides used above. As shown in Figure 3, cells that were treated with EDA^+^ peptide covered the damaged area faster than cells that were cultured in the presence of EDA- peptide or that in the control without any addition. Similar results were obtained in HUES 5 hESC line (Figure S2).

Since the wound healing rate could be the consequence of cell migration, besides proliferation, we next quantified proliferation rates by measuring incorporation of BrdU (Table 2). We also measured the proliferation marker nuclear antigen Ki67 (Table 3). Both treatments, medium supplemented with EDA^+^ peptide and EDA^+/+^MEF-CM increased BrdU incorporation and the percentage of Ki67 positive cells with respect to untreated cells, which were cultured on MEF conditioned medium. These results indicate that EDA increases proliferation rate in the human embryonic stem cell line WAO9. In summary, we found that ESC cultured with CM containing EDA+FN or recombinant peptides containing the EDA show a higher proliferation rate than ESC supplemented with CM from EDA-MEFs or peptides lacking the EDA. This effect was observed in mouse and human ESC lines, as determined by different experimental approaches. In fact, we observed the same effect in Ainv15 mESC line, detected by MTT or crystal violet at different days of treatment, and in WAO9 hESC line, detected by the wound healing assay, BrdU incorporation and Ki67 staining. 

**Table 2:** Analysis of BrdU incorporation in WAO9 hES cells in different media conditions.

	**Positive BrdU cells (%)**	N^(1)^	**p value** ^(2)^
**Control**	29,78	943	
**EDA+/+ MEF-CM**	70,34	1147	<0.001
**FN EDA+ Peptide**	85,70	1038	<0.001

(1) Number of total cells positive for DAPI staining (2) p values resulted from comparison of each treatment with the control. A Chi Square test was used to compare the number of positive BrdU cells between control and each treatment. Bonferroni´s method was used to correct p values for multiple comparisons. Control: untreated cells. Values correspond to a representative experiment with three biological replicates.

**Table 3:** Analysis of Ki67 presence in WAO9 hES cells in different media conditions.

	**Positive Ki67 cells (%)**	N^(1)^	**p value** ^(2)^
**Control**	19,49	1519	
**EDA+/+ MEF-CM**	33,80	1284	<0.001
**FN EDA+ Peptide**	49,26	1011	<0.001

(1) Number of total cells positive for DAPI staining (2) p values resulted from comparison of each treatment with the control. A Chi Square test was used to compare the number of positive Ki67cells between control and each treatment. Bonferroni´s method was used to correct p values for multiple comparisons. Control: untreated cells. Values correspond to a representative experiment with three biological replicates.

**Figure 4:**
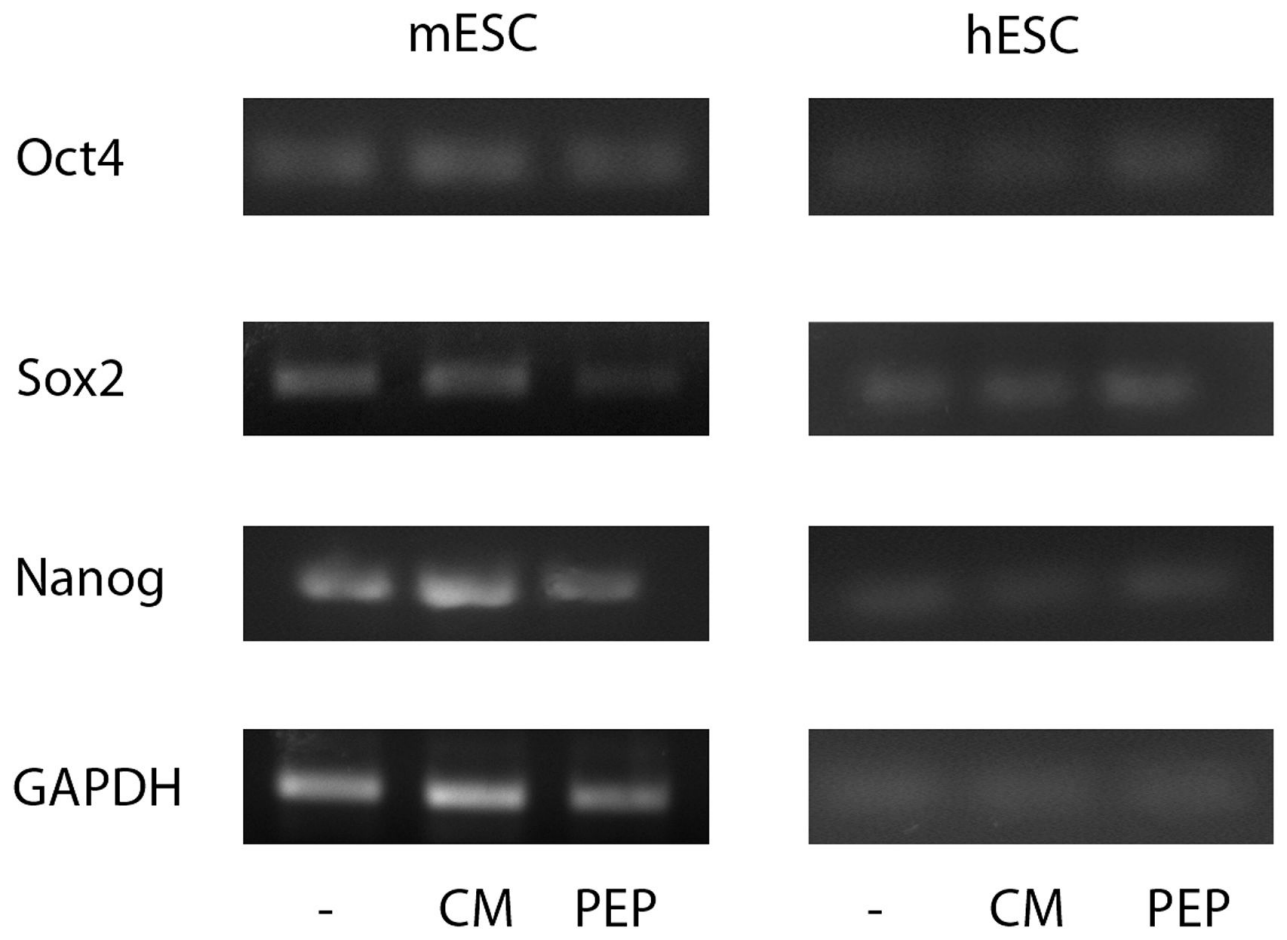
EDA –treated ES cells express pluripotency markers. WA09 human ES cells and Ainv15 mouse ES cells were cultured for three days on feeder free conditions in the indicated medium (untreated cells, C; EDA^+/+^ MEF-CM, CM; EDA+ including peptide, PEP). RNA was extracted and the expression of Oct4, Sox2 and Nanog pluripotency gene markers was analyzed by RT-PCR. The expression of the housekeeping GAPDH gene was used as control.

**Figure 5:**
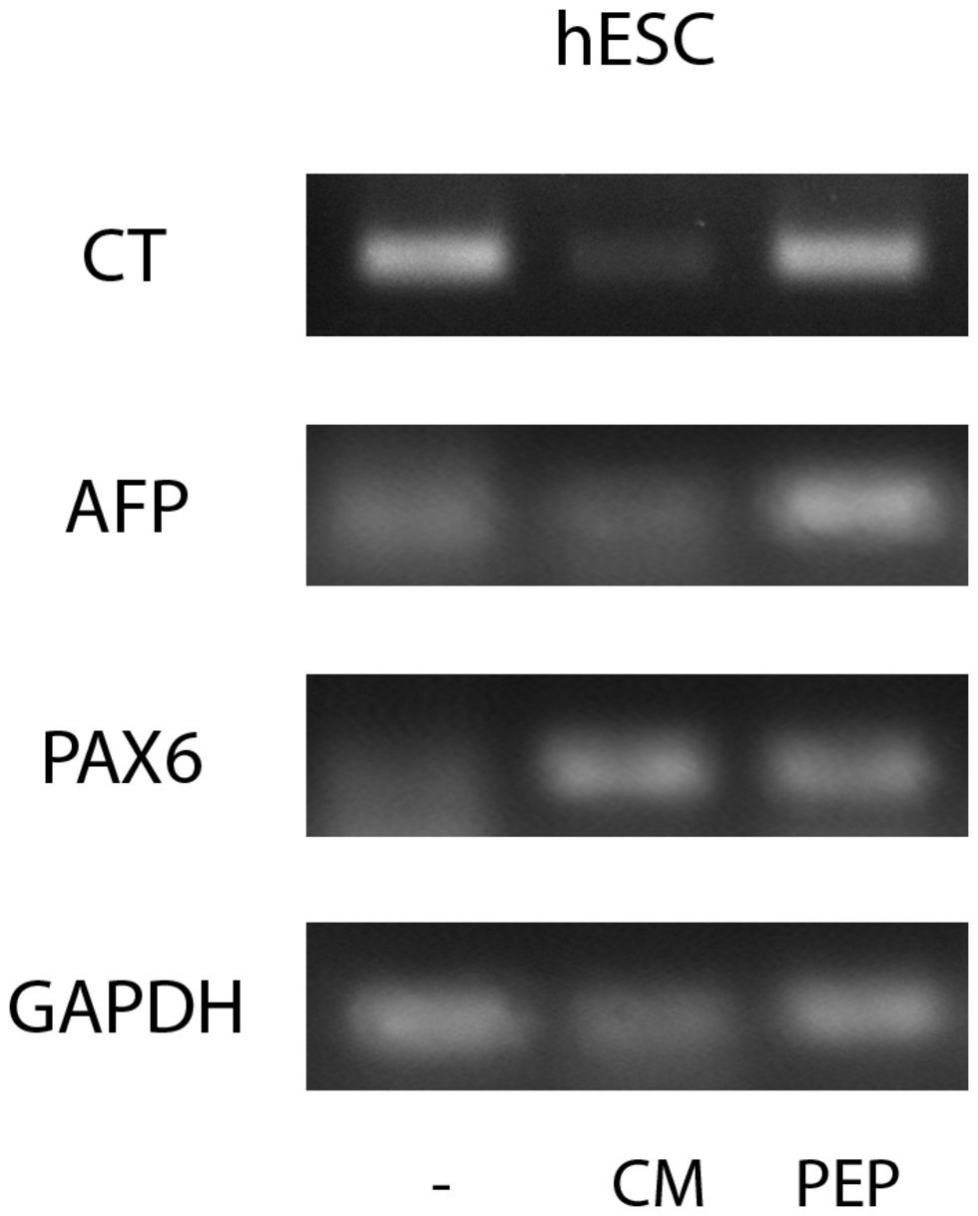
EDA–treated human ESC can differentiate to the three germ layers. WA09 hES cells were cultured for three days on Matrigel coated dishes in the indicated medium (untreated cells, C; EDA^+/+^ MEF-CM, CM; EDA+ including peptide, PEP) and then *in* *vitro* differentiated as described in Material and Methods Section for seven days. RNA was extracted and the expression of lineage specific gene markers was analyzed by RT-PCR. Alpha-fetoprotein (AFP), endoderm gene marker; Cardiac Troponin (CT), mesoderm gene marker; PAX6, ectoderm gene marker.

**Figure 6:**
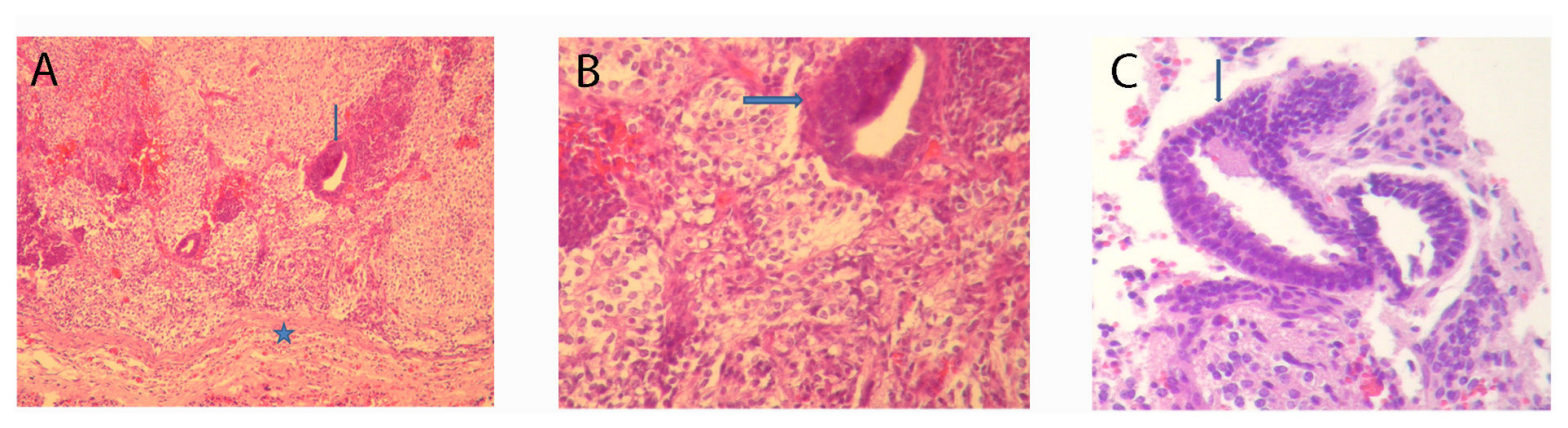
EDA–treated mouse ESC can differentiate to the three germ layers. Ainv15 mES cells were cultured for three days on gelatin coated dishes in the presence of EDA+ peptide. Then, 10^6^ cells were injected subcutaneously into nude mice, as described in Material and Methods Section. Four-micrometer sections from teratoma tissue were stained with hematoxylin and eosin. (A–C) Representative histology of teratoma (A) Neuroepithelium (arrow, ectoderm) and mesenchymal tissue (star, mesoderm); (B) Neuroepithelium (arrow, ectoderm) and glial tissue (ectoderm); Glandular tissue (arrow, endoderm).

Finally, we wondered if ES cells treated with EDA containing FN preserved their basic properties. We evaluated both self-renewal and pluripotency on ES cells cultured for three days in the presence of exogenous EDA. We found that EDA treated-ES cells expressed pluripotency markers Oct4, Sox2 and Nanog (Figure 4). Futhermore, these cells remained pluripotent, as they gave raise to cells deriving from the three germ layers, evidenced by an *in vitro* differentiation protocol and teratoma assay, in human ES cells and mouse ES cells, respectively. (Figure 5 and Figure 6). 

## Discussion

Extracellular matrix components interact with transmembrane cellular receptors belonging to the integrin family and stimulate cell proliferation. One of these extracellular matrix proteins, FN, is highly efficient in cell cycle progression stimulation [28–30]. Until recently, the mechanisms involved in the effect of FN and FN isoforms on stem cells proliferation had not been studied. FN expression in mouse ES cells is modulated by glucose levels in the culture medium and it was suggested that the increased FN synthesis was responsible for the augmented proliferation in response to high glucose concentrations [9]. It was also reported that hypoxia increases proliferation of mES cells via FN and β1 Integrin induction [31]. Unfortunately, a more detailed analysis addressing the FN isoforms involved was not yet performed. Here, we more finely studied the effects on ES cells proliferation of FN addition by determining the FN isoform involved. In fact, we showed that the increase in ESC proliferation rate caused by the addition of FN containing the EDA, an isoform present in embryos and in actively duplicating tissues, was significantly higher than that observed after the addition of FN lacking the EDA, an isoform identical to pFN, which is commonly used as FN source.

It was also shown that FN induced protooncogenes and cell-cycle regulatory proteins mediated by signaling pathways involving RhoA-PI3K/Akt-ERK 1/2 and caveolin-1 in mouse ES cells [32]. It was recently reported that in mES cells cultured in feeder-free conditions, fibronectin produced by mES cells or added to the medium, interacts with the gelatin substrate, providing a surface that supports cell adhesion and self-renewal [33].

Previously, it had been shown that the FN isoform that includes the extra domain A was mitogenic for granulosa cells [15]. Contrary to plasma FN, which is synthesized in the liver and lacks this exon, FN EDA^+^ is highly expressed in embryonic tissues and inclusion of the EDA domain decreases with aging [34–36]. FN EDA^+^ is found in proliferating tissues, wound healing [17,37] and tissue fibrosis [18,38,39]. It was also reported that this isoform stimulates keratinocyte hyperproliferation [40] and lung fibroblast proliferation [41], and that it is related with tumor malignancy [42–44] and psoriasis [40]. In addition, it was shown that FN EDA^+^ induces cyclin D1 expression, pRb hyperphosphorylation, and extracellular signal regulated kinase 2 activation [19]. These evidences as a whole strongly support that this isoform is involved in cell proliferation. 

In this work we found that FN EDA^+^ is a mitogenic factor for mouse and human ESC. We addressed this question by two different experimental approaches. We provided FN EDA^+^ or FN EDA^-^ to ES cells by culturing them in media that was previously conditioned by genetically modified MEFs only secreting FN EDA^+^ or FN- EDA^-^, or in standard culture medium with the addition of recombinant peptides containing or not the EDA. With both approaches we could appreciate the higher proliferation rate of ES cells cultured in the presence of EDA. We found a high decrease in proliferation when cells were cultured in EDA^-/-^ MEF-CM respect to *wt* MEF-CM or EDA^+/+^ MEF-CM. As the EDA^-/-^ MEF line does not express FN containing EDA [23], the lower proliferation rate in cells cultured with EDA^-/-^ MEF-CM is apparently related to the absence of EDA-containing FN in the medium. Notably, although we noticed a slight increase in proliferation in EDA^+/+^ MEF-CM respect to *wt* MEF-CM, the differences between these two conditions were not so pronounced. It is probable that the levels of FN EDA^+^ present in *wt* MEF-CM could be high enough to promote mES cells proliferation.

Otherwise, mouse and human ES cells endogenously express EDA containing FN (Figure S3). We hypothesized that the levels of endogenous EDA modulate the magnitude of the response to exogenous EDA. We are studying this hypothesis by modulation of endogenous FN EDA^+^.

Regarding the FN receptors, even though cells normally express a wide repertoire of integrins, and that they exhibit a high level of promiscuity of their ligands, the α5/β1 integrin is considered as the classic FN receptor, since it possesses high binding-affinity only to this protein [45,46]. Different integrin receptors have been reported to bind the EDA: the α4β7 integrin partially mediates adhesion to FN EDA^+^ in murine and human lung fibroblasts [24], α4β1 was reported as EDA receptor in human melanoma cells [47], and α9β1 integrin was also proposed as the FN EDA^+^ specific receptor in multiple models [42,47–50]. However, to our knowledge, there is no evidence so far about the specific FN EDA^+^ receptor in stem cells. Further work should be done to determine if the same receptors and effectors that mediate the different FN EDA^+^ effects on other cell types are responsible for increasing mouse and human ES cells’ proliferation rate. 

With respect to human ES cells, extracellular matrix proteins have been extensively studied as scaffold or substrate to both promote undifferentiated propagation by activation of adhesion and signaling pathways responsible for their self-renewal [51], and to direct differentiation [52]. However, to our knowledge this is the first report showing that a particular soluble FN isoform increases hES cells’ proliferation when added to the culture medium.

In summary, we reported that FN EDA^+^ increases proliferation in mouse and human ESC. We are currently studying if the same phenomenon is observed when culturing iPSCs and the molecular mechanisms involved. Elucidating the actors implicated in stem cells proliferation would contribute to the understanding of pluripotent stem cells biology.

## Supporting Information

Figure S1 **Dose-response curve.** Ainv15 mES cells were plated in standard proliferation medium. 24 hours later, when the cells were attached, medium was replaced by fresh medium containing the corresponding peptide. (A) Dose-response curve. ES cells were cultured in the standard media supplemented by progressive dilutions of EDA-containing or EDA-lacking recombinant peptide preparations (EDA^+^ or EDA^-^, respectively), as indicated, for 72 hours. Dilution 1 corresponds to peptide preparation containing 10 µg of protein per ml of medium. Proliferation was evaluated by crystal violet staining assay. A representative experiment of at least three replicates is shown. Data are shown as mean ± SD. Statistical analysis was done by Two way ANOVA with Bonferroni test for multiple comparisons. Different uppercase letters indicate significant differences between dilutions (*P* < 0.01). Different lowercase letters indicate significant differences between EDA treatments (*P* < 0.001).
(TIF)

Figure S2 **EDA^+^ but not EDA^-^ peptide increases the proliferation rate of HUES-5 hESC.** HUES-5 human ES cells were plated in standard proliferation medium. Twenty four hours later, when the cells were attached, medium was replaced by fresh medium containing or not (-) the corresponding peptide preparation to a final dose of 10 µg of protein per ml of medium; EDA-containing or EDA-lacking recombinant peptides (EDA^+^ or EDA^-^, respectively). (A) Representative brightfield pictures of the wound-healing assay. The scratch was made the same day that the peptide was added to the medium. (B) Quantification of the filled areas of a representative experiment of three replicates. Proliferation was calculated from the decrement in damaged area. The areas were quantified with the ImageJ software. The filled area in each condition was referred as the amount of filled area in the control, considered as 100%. The graph is representative of a scratch wound healing assay of three replicates.
(TIF)

Figure S3 **Mouse and human ES cells express FN EDA+.** WA09 human ES cells and Ainv15 mouse ES cells were cultured in standard proliferation media. RNA was extracted and the expression of FN EDA+ was analyzed by RT-PCR. The expression of the housekeeping GAPDH gene was used as control.
(TIF)

Table S1 **Gene Specific Primers used in Reverse Transcription–Polymerase Chain Reaction.**
(DOCX)
