# Supplementary figures and images for: EDA-Containing Fibronectin Increases Proliferation of Embryonic Stem Cells
Source: PLoS One. 2013 Nov 14;8(11):e80681. doi: 10.1371/journal.pone.0080681 (PMC3828241; doi:10.1371/journal.pone.0080681)

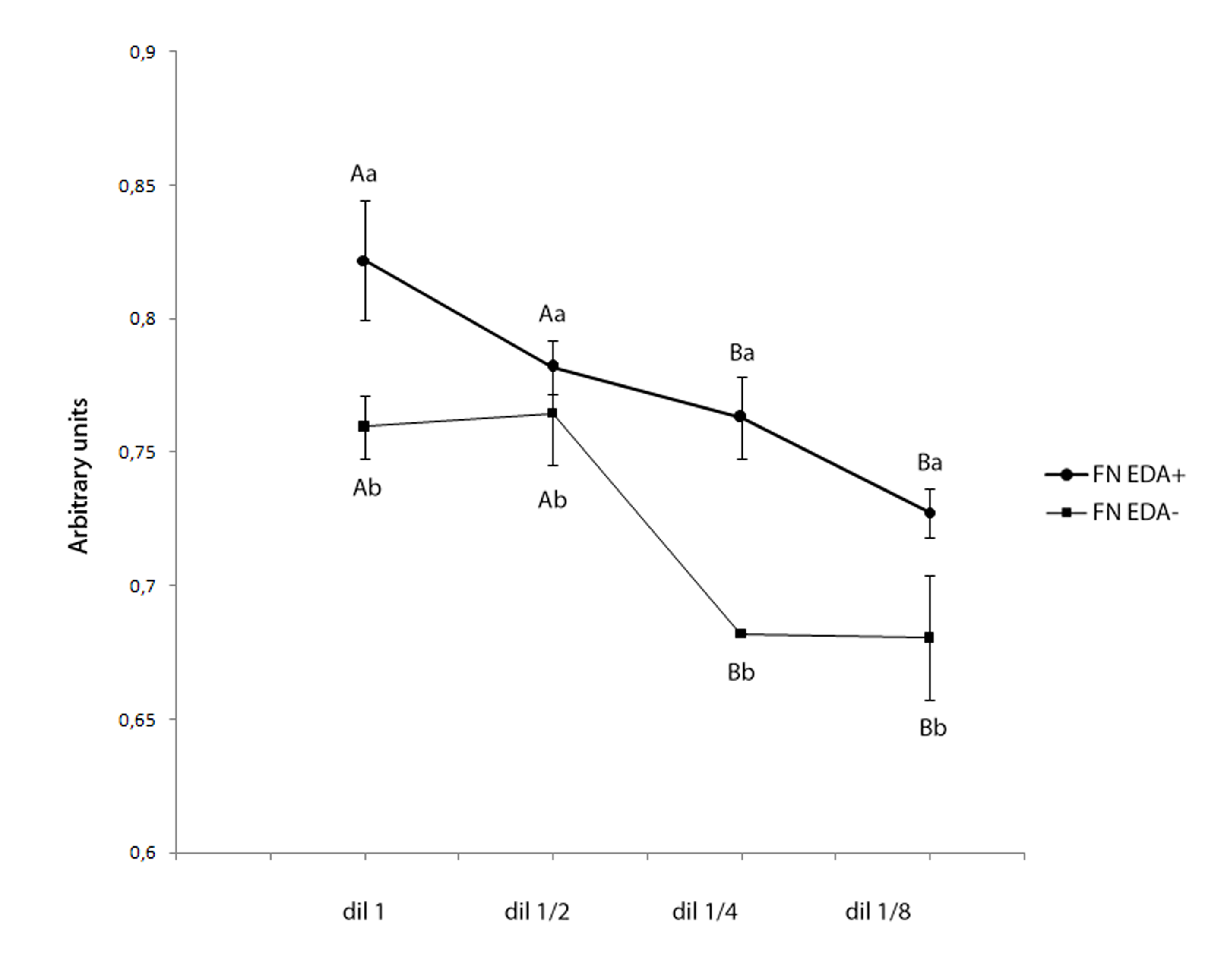

Supplement: Figure S1 — Dose-response curve. Ainv15 mES cells were plated in standard proliferation medium. 24 hours later, when the cells were attached, medium was replaced by fresh medium containing the corresponding peptide. (A) Dose-response curve. ES cells were cultured in the standard media supplemented by progressive dilutions of EDA-containing or EDA-lacking recombinant peptide preparations (EDA+ or EDA-, respectively), as indicated, for 72 hours. Dilution 1 corresponds to peptide preparation containing 10 µg of protein per ml of medium. Proliferation was evaluated by crystal violet staining assay. A representative experiment of at least three replicates is shown. Data are shown as mean ± SD. Statistical analysis was done by Two way ANOVA with Bonferroni test for multiple comparisons. Different uppercase letters indicate significant differences between dilutions (P < 0.01). Different lowercase letters indicate significant differences between EDA treatments (P < 0.001). (TIF) [file pone.0080681.s001.tif]

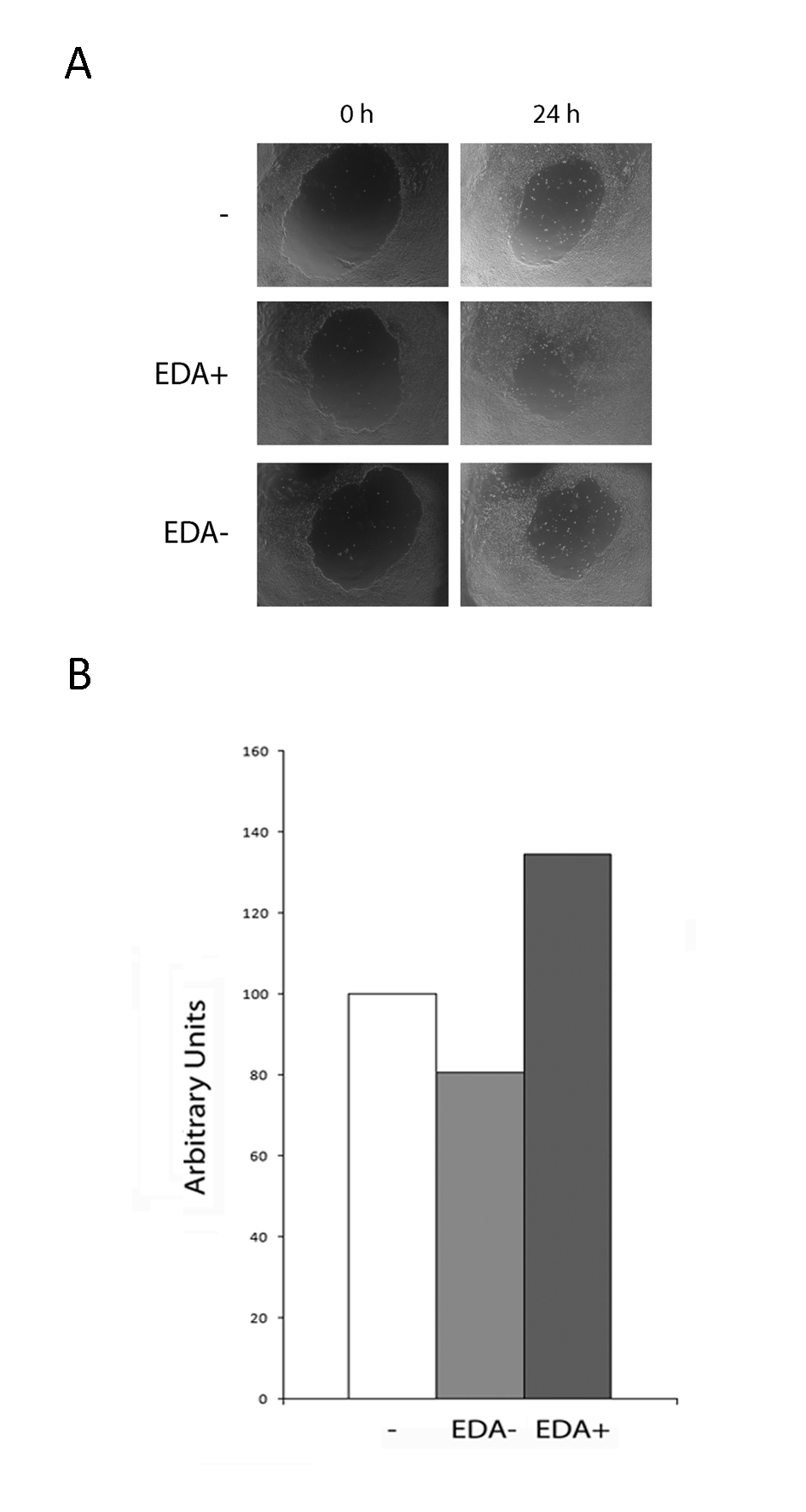

Supplement: Figure S2 — EDA+ but not EDA- peptide increases the proliferation rate of HUES-5 hESC. HUES-5 human ES cells were plated in standard proliferation medium. Twenty four hours later, when the cells were attached, medium was replaced by fresh medium containing or not (-) the corresponding peptide preparation to a final dose of 10 µg of protein per ml of medium; EDA-containing or EDA-lacking recombinant peptides (EDA+ or EDA-, respectively). (A) Representative brightfield pictures of the wound-healing assay. The scratch was made the same day that the peptide was added to the medium. (B) Quantification of the filled areas of a representative experiment of three replicates. Proliferation was calculated from the decrement in damaged area. The areas were quantified with the ImageJ software. The filled area in each condition was referred as the amount of filled area in the control, considered as 100%. The graph is representative of a scratch wound healing assay of three replicates. (TIF) [file pone.0080681.s002.tif]

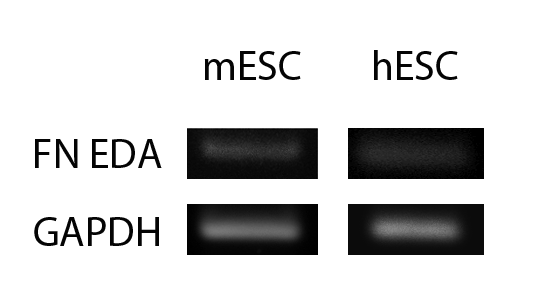

Supplement: Figure S3 — Mouse and human ES cells express FN EDA+. WA09 human ES cells and Ainv15 mouse ES cells were cultured in standard proliferation media. RNA was extracted and the expression of FN EDA+ was analyzed by RT-PCR. The expression of the housekeeping GAPDH gene was used as control. (TIF) [file pone.0080681.s003.tif]
