# Supplementary material for: EDA-Containing Fibronectin Increases Proliferation of Embryonic Stem Cells
Source: PLoS One. 2013 Nov 14;8(11):e80681. doi: 10.1371/journal.pone.0080681 (PMC3828241; doi:10.1371/journal.pone.0080681)
Supplement: Table S1 — Gene Specific Primers used in Reverse Transcription–Polymerase Chain Reaction. (DOCX) [file pone.0080681.s004.docx]

**Table S1.** Gene Specific Primers used in Reverse Transcription–Polymerase Chain Reaction

| *Gene Name* |  | *Primer sequence (5’-3´)* |
| --- | --- | --- |
| Human  FN EDA+ | Forward  Reverse | CCGGGTTCTGAGTACACAGTC  AGTTGGTGCAGGAATAGCTG |
| Mouse  FN EDA+ | Forward  Reverse | CAGAAATGACCATTGAAGGT  ATGAGTCCTGACACAATCAC |
| Human  Cardiac troponin | Forward  Reverse | ATGATGCATTTTGGGGGTTA  CAGCACCTTCCTCCTCTCAG |
